# Comparative Transcriptomic Analyses of Differentially Expressed Genes in Transgenic Melatonin Biosynthesis Ovine *HIOMT* Gene in Switchgrass

**DOI:** 10.3389/fpls.2016.01613

**Published:** 2016-11-08

**Authors:** Shan Yuan, Cong Guan, Sijia Liu, Yanhua Huang, Danyang Tian, Xin Cui, Yunwei Zhang, Fuyu Yang

**Affiliations:** ^1^College of Animal Science and Technology, China Agricultural UniversityBeijing, China; ^2^College of Agriculture, China Agricultural UniversityBeijing, China; ^3^Beijing Key Laboratory for Grassland Science, China Agricultural UniversityBeijing, China; ^4^National Energy R&D Center for BiomassBeijing, China; ^5^Beijing Sure Academy of BiosciencesBeijing, China

**Keywords:** switchgrass, melatonin, transgene, *oHIOMT*, RNA-seq

## Abstract

Melatonin serves pleiotropic functions in prompting plant growth and resistance to various stresses. The accurate biosynthetic pathway of melatonin remains elusive in plant species, while the *N*-acetyltransferase and *O*-methyltransferase were considered to be the last two key enzymes during its biosynthesis. To investigate the biosynthesis and metabolic pathway of melatonin in plants, the RNA-seq profile of overexpression of the ovine *HIOMT* was analyzed and compared with the previous transcriptome of transgenic *oAANAT* gene in switchgrass, a model plant for cellulosic ethanol production. A total of 946, 405, and 807 differentially expressed unigenes were observed in *AANAT* vs. control, *HIOMT* vs. control, and *AANAT* vs. *HIOMT*, respectively. Two hundred and seventy-five upregulated and 130 downregulated unigenes were detected in transgenic *oHIOMT* line comparing with control, including the significantly upregulated (F-box/kelch-repeat protein, zinc finger BED domain-containing protein-3) genes, which were potentially correlated with enhanced phenotypes of shoot, stem and root growth in transgenic *oHIOMT* switchgrass. Several stress resistant related genes (SPX domain-containing membrane protein, copper transporter 1, late blight resistance protein homolog R1A-6 OS etc.) were specifically and significantly upregulated in transgenic *oHIOMT* only, while metabolism-related genes (phenylalanine-4-hydroxylase, tyrosine decarboxylase 1, protein disulfide-isomerase and galactinol synthase 2 etc.) were significantly upregulated in transgenic *oAANAT* only. These results provide new sights into the biosynthetic and physiological functional networks of melatonin in plants.

## Introduction

Melatonin (*N*-acetyl-5-methoxytryptamine) was first discovered in vertebrates (Lerner et al., 1958), then detected in higher plants (Dubbels et al., 1995; Hattori et al., 1995), and now is widely accepted of its distribution in all kingdoms, from prokaryotes to eukaryotes, from animals to plants (Manchester et al., 2000; Hardeland and Poeggeler, 2003; Simopoulos et al., 2005; Hardeland, 2015), and functions as a direct scavenger of reactive oxygen species (ROS), a mediator hormone of circadian rhythms and an activator of antioxidant enzymes (Arnao and Hernández-Ruiz, 2015; Reiter et al., 2015; Bai et al., 2015, 2016; Gao et al., 2016; Shi et al., 2016a). In plants, melatonin regulated the seed germination, growth of roots, and shoots, and development of flowering (Hernández-Ruiz et al., 2005; Zhao et al., 2013; Arnao and Hernández-Ruiz, 2015). Moreover, the responses to abiotic stresses, including extreme temperature, drought, salt, radiation, make melatonin a concerned candidate as a natural stimulator for crop cultivation (Li et al., 2012; Zhang et al., 2014a; Fan et al., 2015; Shi et al., 2015a). However, the involved regulations of the functional gene expression and physiological mechanism of melatonin biosynthesis and metabolic pathways remains poorly understood in plants (Hardeland, 2015; Zhang et al., 2014b).

The classic pathway of melatonin biosynthesis comprises four steps beginning with tryptophan, firstly decarboxylation by tryptophan decarboxylase (TDC), then *N*-acetylation by arylalkylamine *N*-acetyltransferase (AANAT) in animals/serotonin *N*-acetyltransferase (SNAT) in plants, and the final *O*-methylation by *N*-aceylserotonin methyltransferase (ASMT) in animal/hydroxyindole-*O*-methyltransferase (HIOMT) in plants (Tan et al., 2016). The last two steps are presumed as rate-limiting which are catalyzed by AANAT/SNAT and HIOMT/ASMT (Morton and Forbes, 1988; Byeon et al., 2015, 2016). Furthermore, the overexpression of rice caffeic acid *O*-methyltransferase (*COMT*) exhibited upregulation of melatonin contents in transgenic rice plants, indicating that the *N*-acetylserotonin methyltransferase activity was required for melatonin biosynthesis (Byeon et al., 2015). Recently, genetic engineering modifications of coding genes for melatonin biosynthesis and metabolism enzymes were applied to alter the melatonin contents in transgenic rice, tomato, *Arabidopsis thaliana*, and *Nicotiana sylvestris* (Kang et al., 2010; Okazaki et al., 2010; Park et al., 2012; Zhang et al., 2012; Wang et al., 2014; Zuo et al., 2014).

Switchgrass (*Panicum virgatum*) is a Poaceae warm season C4 perennial grass native to the U.S., and is regarded as a model plant of cellulosic biofuel production for its desirable characteristics, such as large biomass and strong ability to thrive in marginal areas (McLaughlin and Kszos, 2005; Keshwani and Cheng, 2009; Brown et al., 2016). Molecular breeding has made much progress toward improving biomass yield, biofuel quality, and stress resistance in switchgrass, which is the model plant for cellulose ethanol production (Bouton, 2007; Fu et al., 2011; Shen et al., 2013; Poovaiah et al., 2014; Wuddineh et al., 2015). Recently, a series of transgenic overexpression of transcriptional factor (*NAC, AP2*/*ERF, MYB4*), sucrose synthesis gene (*SUS*) to prompt growth (Shen et al., 2013; Poovaiah et al., 2014; Yang et al., 2014; Wuddineh et al., 2015), the inhabitation of cinnamyl alcohol dehydrogenase (*CAD*) to reduce the lignin content (Fu et al., 2011), and the exploration of abiotic stress related genes and miRNA (Sun et al., 2012; Sharma et al., 2015; Liu et al., 2016) were produced in switchgrass. In addition, the activities of antioxidant and free radical scavenger for melatonin provide opportunities for prompting growth and development in plants (Arnao and Hernández-Ruiz, 2015; Reiter et al., 2015; Shi et al., 2016a). The exogenous applications of melatonin exhibited the enhanced seed germination in cucumber (Zhang et al., 2014a), lateral root formation in both *Brassica juncea* (Chen et al., 2009) and cucumber (Zhang et al., 2014c), and salt resistance in soybean plants (Wei et al., 2015) etc. Other studies revealed that melatonin also exercised some control over root architecture as observed in St. John's Wort, wild leaf mustard, sweet cherry root stocks, and lupin (Murch et al., 2001; Arnao and Hernández-Ruiz, 2007; Chen et al., 2009; Sarropoulou et al., 2012). Moreover, the endogenous modifications of related genes to gain the melatonin-rich plants displayed cold resistance in rice (Kang et al., 2010), the drought-tolerant phenotypes of tomato (Wang et al., 2014) and *A. thaliana* (Zuo et al., 2014). Here, the *HIOMT* gene encoding the last enzyme in melatonin biosynthesis pathway was overexpressed in switchgrass, and the transcriptomic profile was analyzed in order to disentangle the melatonin biosynthesis pathway and also the potential functions of melatonin in plants.

## Materials and methods

### Plant materials and morphological traits

Transgenic switchgrass (cultivar Alamo) lines expressing ovine *HIOMT* gene (ID: JF815374.1) were grown under 16 h light (26°C, 120 μmol/m^2^/s) and 8 h dark (18°C) conditions in greenhouse. Fully matured plants were chosen from each genotype for molecular characterization and transcriptome sequencing. Three replicates of stems for each transgenic line and in total six (three transgenic *HIOMT* (H) lines: H1, H2, H3, and three transgenic empty vector (EV): EV1, EV2, and EV3) were collected and frozen in liquid nitrogen and stored at 80°C until analysis. The *t*-test was applied to compare the differences of the morphological traits between transgenic *oHIOMT* lines and control.

**Figure 1:**
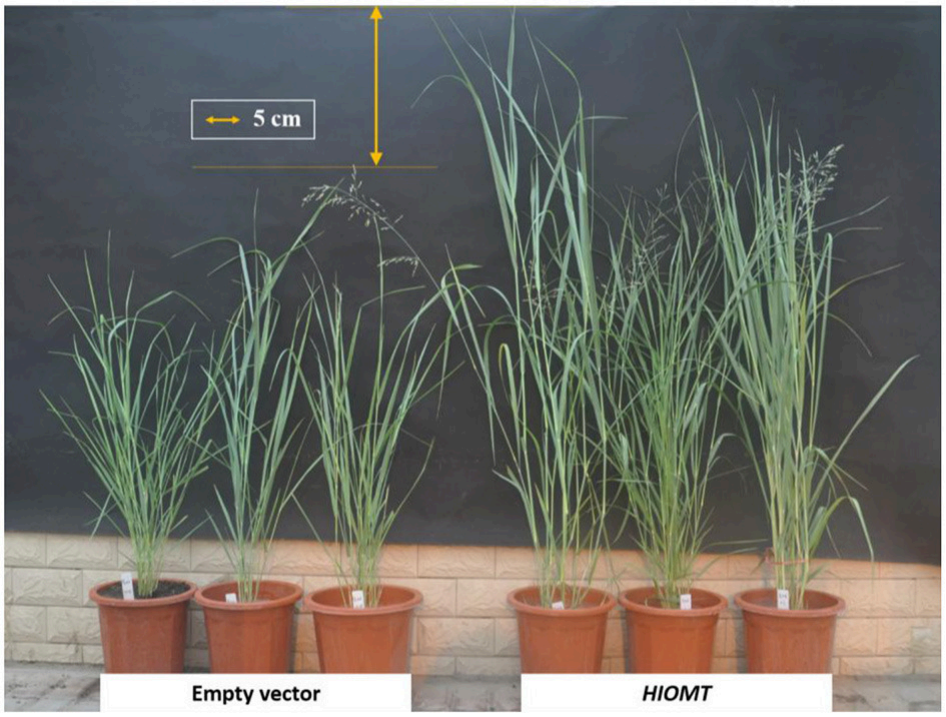
**Phenotypes of the transgenic ***HIOMT*** switchgrass comparing with the transgenic empty vector (EV)**.

At the transgenic reproductive third (R3) stage (Hardin et al., 2013), the tiller number, plant height, stem node number, internode length, internode diameter, leaf blade length, leaf blade width, root number, root length, root diameter, and spike length were determined (Figure 1). The third internode was chosen for measuring internode diameter. Leaf blade length and leaf blade width of third internode were measured. Twelve replicates were randomly sampled for each transgenic line.

### RNA isolation and qualification

Total RNA was extracted from the sampling stems using Trizol method (Invitrogen, Carlsbad, CA, USA). RNA purity and integrity were assessed using the NanoPhotometer® spectrophotometer (IMPLEN, CA, USA) and Agilent Bioanalyzer 2100 system (Agilent Technologies, CA, USA), respectively. RNA concentration was measured using Qubit® RNA Assay Kit in Qubit® 2.0 Flurometer.

### Transcriptome preparation

The 1.5 μg RNA per sample was prepared for the RNA-seq. Sequencing libraries were generated using NEBNext® Ultra™ RNA Library Prep Kit for Illumina® (NEB, USA). The cDNA fragments of 150~200 bp were selected from the library by purification with AMPure XP system (Beckman Coulter, Beverly, USA).

### Clustering

The clustering of the index-coded samples was performed on a cBot Cluster Generation System using TruSeq PE Cluster Kit v3-cBot-HS (Illumia). After cluster generation, paired-end reads were generated by sequencing with the library preparations on an Illumina Hiseq platform.

### Validation of RNA-seq data by real-time quantitative

RNA-seq results were validated by Real-time quantitative PCR of 16 different genes using 7500 Real-Time PCR System (Applied Biosystems) (primer sequences please see Supplementary Table 1). Gene expression levels were calculated by the 2-ΔΔCt method (Livak and Schmittgen, 2001). Each plate was repeated three times in independent runs for all reference and selected genes.

### Data analysis

#### Quality control

After removing the adapter containing reads, ploy-N containing reads and low quality reads, clean data/clean reads were obtained from raw data. Meanwhile, the Q20, Q30 values, GC-contents, and sequence duplication level of the clean data were calculated (Supplementary Table 2). The RNA-seq raw data have been submitted to NCBI SRA dataset (http://www.ncbi.nlm.nih.gov/bioproject), and the project accession number is PRJNA322585.

#### Transcriptome assembly and gene functional annotation

*De novo* transcriptome assembly was constructed and accomplished for the incomplete genome of switchgrass using Trinity with min_kmer_cov set to 2 and all other parameters set default (Grabherr et al., 2011). Gene function was annotated based on the following databases: Nr (NCBI non-redundant protein sequences); Nt (NCBI non-redundant nucleotide sequences); Pfam (Protein family); KOG/COG (Clusters of Orthologous Groups of proteins); Swiss-Prot (A manually annotated and reviewed protein sequence database); KO (KEGG Ortholog database); GO (Gene Ontology).

#### Differential expression analysis

Gene expression levels were calculated by RSEM (Li and Dewey, 2011). To reveal the transcriptionally regulatory event occurring during the transgenic process, comparative transcriptomic analysis was performed among the pools of control and the two transgenic (o*AANAT* and o*HIOMT*) RNA samples. Only the genes with a *P*-value-adjusted (padj) < 0.05 were identified as being significantly changed by the Benjamini and Hochberg's approach.

#### GO enrichment analysis and KEGG pathway enrichment analysis

Gene Ontology (GO) enrichment analysis of the differentially expressed genes (DEGs) was implemented by the GOseq R packages based Wallenius non-central hyper-geometric distribution (Young et al., 2010), which can adjust for gene length bias in DEGs. KEGG (Kanehisa et al., 2008) is a database resource for understanding high-level functions and utilities of the biological system, such as the cell, the organism, and the ecosystem, from molecular-level information, especially large-scale molecular datasets generated by genome sequencing and other high-throughput experimental technologies (http://www.genome.jp/kegg/). The KOBAS software was performed to test the statistical enrichment of differential expression genes in KEGG pathways (Mao et al., 2005).

## Results

### Promoted vegetative and reproductive growth

To compare the effects of *HIOMT* gene during the biosynthesis processes, the phenotypic traits were measured. Transgenic *HIOMT* switchgrass exhibited significant promotion of growth comparing with EV (Figure 1; Supplementary Table 3). Average plant height and internode length were 35.6 and 52.9% higher in *HIOMT* (90.60 and 13.43 cm) than that of EV (66.81 and 8.78 cm, respectively, *P* < 0.05). Stem node number of *HIOMT* was 15.6% higher than that of EV (*P* > 0.05). There was no significant difference in internode diameter between the two groups (*P* > 0.05). Average leaf blade length was 20.7% longer in *HIOMT* (52.53 cm) than that of EV (43.53 cm, *P* < 0.05). Root number, root length and root diameter of *HIOMT* were 75.8, 18.3, and 39.4% larger than those of EV, respectively (*P* < 0.05). Spike length in *HIOMT* (19.5 cm) was nearly 4-fold of the EV (5.4 cm, *P* < 0.01).

### Differential expression profiling between transgenic o*HIOMT* and EV lines

**Figure 2:**
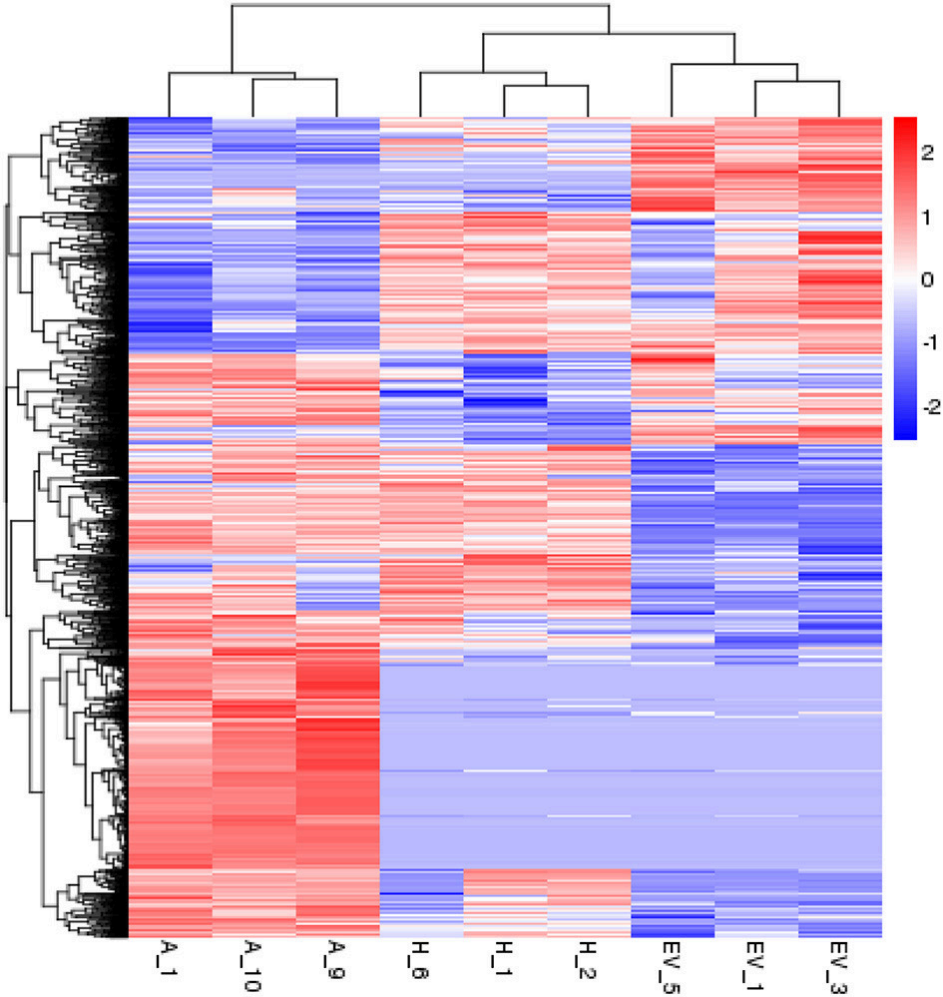
**Hierarchical clustering of the differentially expressed genes, using the RNA-seq data derived from three groups (A: ***AANAT***, H: ***HIOMT***, and EV: empty vector, hereafter) based on log10 RPKM values**.

**Figure 3:**
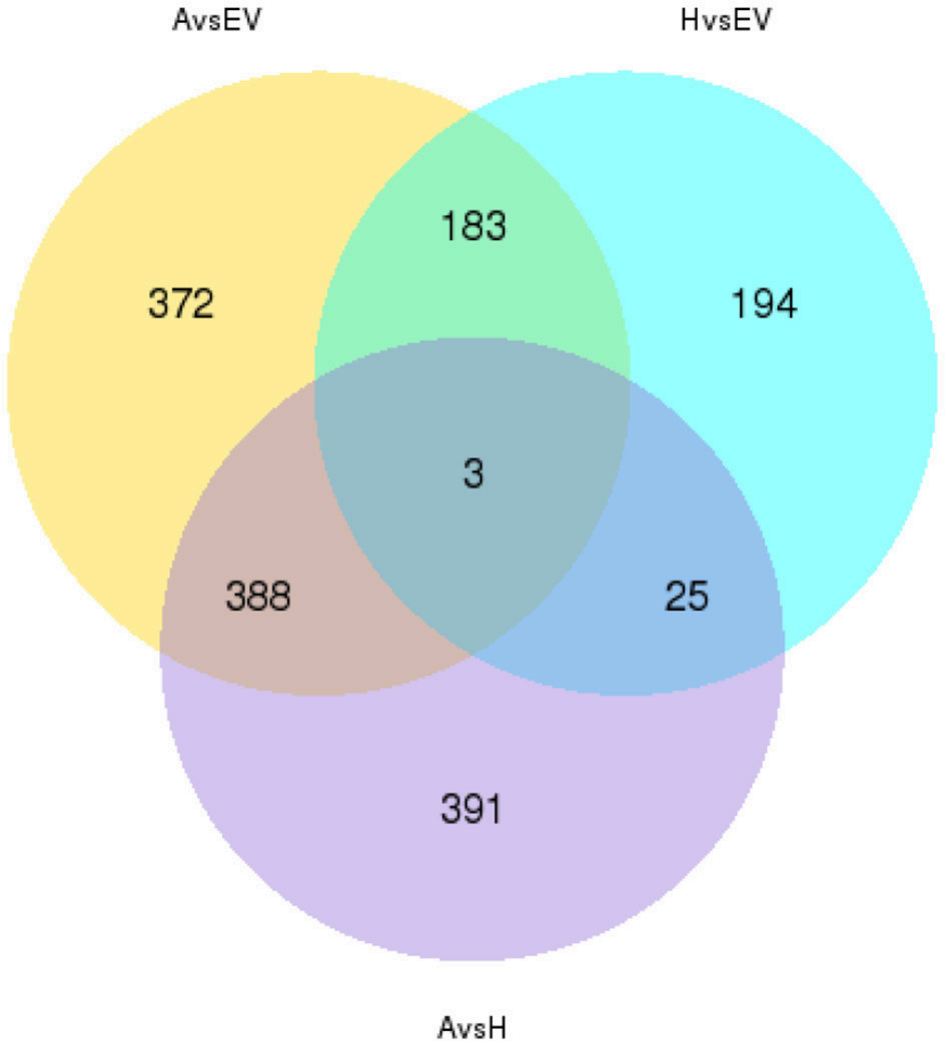
**Venn diagram showing the number of differentially expressed genes between every two samples and the number of joint differentially expressed genes**.

To analyze the similarities and differences in the transgenic transcriptome, a hierarchical clustering was represented and indicated the significant differences in transcripts of all of the DEGs in the three replicates of the control and two transgenic groups (Figure 2). After calculating the expression level of each mapped unigene, a total of 1556 unigenes were detected that had levels of expression that were significantly different among the two transgenic and control libraries. The 946, 405, and 807 differentially expressed unigenes were observed in *AANAT* vs. EV, *HIOMT* vs. EV, and *AANAT* vs. *HIOMT*, respectively (Figure 3). A total of 183 genes were differentially expressed in both *AANAT* vs. EV and *HIOMT* vs. EV, but not significantly different in *AANAT* vs. *HIOMT*. Three unigenes overlapped with all three groups, including c52804_g3 (hypothetical protein), c56995_g3 (*O*-methyltransferase), c56995_g4 (ribosomal protein). One hundred twenty nine unigenes were upregulated in both transgenic *oAANAT* and *oHIOMT* comparing with EV, while 55 unigenes were downregulated. There were 145 and 76 genes that significantly upregulated and downregulated in transgenic *oHIOMT*, respectively, but not differentially expressed in transgenic *oAANAT*. Comparatively, there were 607 and 153 genes that significantly upregulated and downregulated in transgenic *oAANAT*, while not differentially expressed in transgenic *oHIOMT* (Supplementary File 1). Twenty-five unigenes specifically and differentially expressed in *HIOMT*, including 13 upregulated (c64524_g1, c52718_g1: glycine-rich domain-containing protein 1-like; c43728_g1: Bowman-Birk type wound-induced proteinase inhibitor WIP1-like; c62742_g1: SPX domain-containing membrane protein) and 12 downregulated ones (c46269_g1: NAC domain-containing protein 67-like; c61012_g1: protein tyrosine kinase) comparing with *AANAT* and EV (Supplementary File 1).

### Functional classification of the DEGs by gene ontology analysis

**Figure 4:**
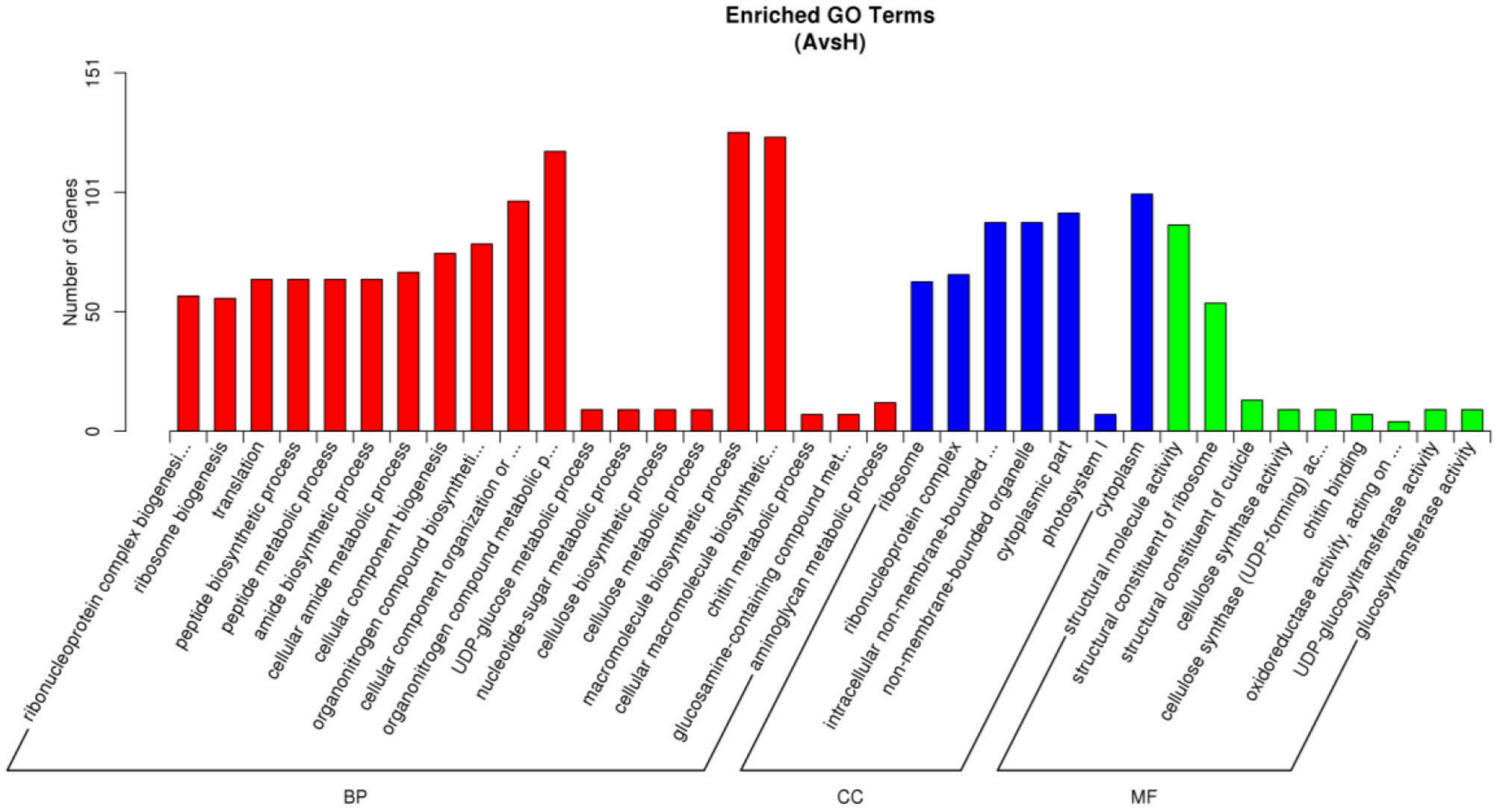
**GO classifications of DEGs between transgenic ***AANAT*** and ***HIOMT*** lines in three main categories: biological process (BP), cellular component (CC), and molecular function (MF)**.

Among all differentially expressed unigenes, 1071 were upregulated among the three libraries, and 513 were downregulated. However, only 511, 193, and 504 unigenes were functionally annotated with GO terms in *AANAT* vs. EV, *HIOMT* vs. EV, and *AANAT* vs. *HIOMT*, respectively, revealed by DEG analysis were functionally assigned to the relevant terms in three categories (Biological Process, Cellular Component, and Molecular Function) of the GO database (Figure 4). The GO terms “organonitrogen compound biosynthetic process,” “oxidoreductase activity,” “NAD(P)H oxidase activity,” growth factor activity, and “hyperosmotic response” were highly enriched in the DEGs. At the same time, other terms that were related to the response to various other types of abiotic and biotic stresses, such as SOS response “phototropism,” “cellular response to external stimulus,” and “response to external stimulus,” were also highly enriched in the DEGs.

By comparing transgenic *AANAT* vs. EV, 2012 GO terms were DEGs, including 1687 upregulated and 1094 downregulated GO items. The GO terms of the “peptide metabolic process” and “amide biosynthetic process” in Biological Process and “structural molecule activity” in Molecular Function were significantly overrepresented (Figure 4). By comparing transgenic *HIOMT* vs. EV, 1314 DEGs, including 724 downregulated GO items and 1062 upregulated GO items, were functionally assigned to the relevant terms; “metabolic process” in Biological Process was significantly overrepresented, followed by “oxidation-reduction process” in Biological Process and “oxidoreductase activity” in Molecular Function. Notably, none of the GO terms was significantly enriched after multiple testing corrections (corrected *P* > 0.05) in *HIOMT* vs. EV. Considering the *AANAT* vs. *HIOMT*, 1904 DEGs, including 1493 upregulated and 1126 downregulated GO items, were significantly overrepresented of the “macromolecule biosynthetic process” and “cellular macromolecule biosynthetic process” in Biological Process, “cytoplasm,” and “cytoplasmic part” in Cell Component, and “structural molecule activity” and “structural constituent of ribosome” in Molecular Function (Figure 4).

### KEGG pathway analysis of the melatonin-related genes

**Figure 5:**
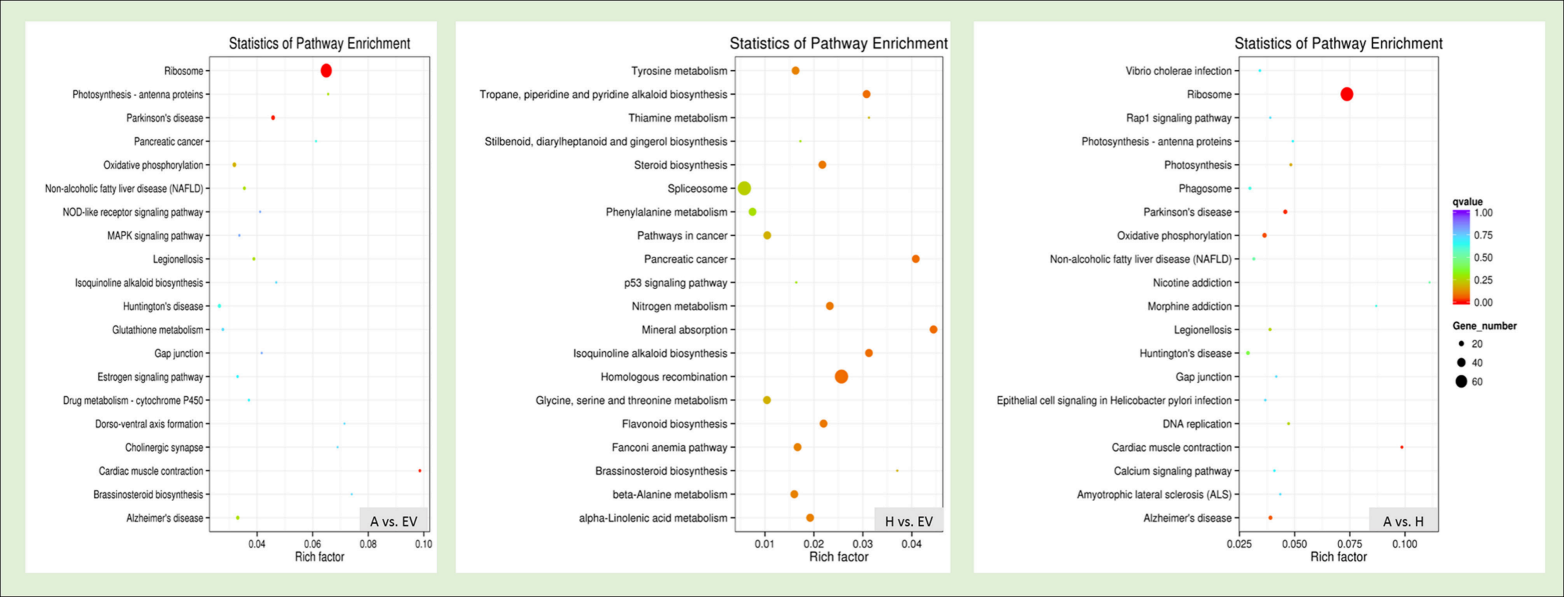
**KEGG enrichments of the annotated DEGs across three comparisons**. The left Y-axis indicates the KEGG pathway. The X-axis indicates the Rich factor. A high *q*-value is represented by blue, and a low *q*-value is represented by red.

The 50 pathways were identified between the *HIOMT* vs. EV from the KEGG database, with 36 upregulated and 17 downregulated pathways (Figure 5). The DEGs were significantly enriched in “homologous recombination,” “mineral absorption,” “isoquinoline alkaloid biosynthesis,” “tropane, piperidine, and pyridine alkaloid biosynthesis,” “nitrogen metabolism,” “flavonoid biosynthesis,” “steroid biosynthesis,” “alpha-Linolenic acid metabolism,” “tyrosine metabolism,” and “beta-Alanine metabolism.” The top 20 obviously enriched pathways are shown in Figure 5. Specifically, the photosynthesis-antenna proteins pathway, MAPK signaling pathway and oxidative phosphorylation were significantly upregulated in KEGG pathway maps during the transgenic *oAANAT* line. While nitrogen metabolism, flavonoid biosynthesis, beta-Alanine metabolism, Brassinosteroid biosynthesis, Phenylalanine metabolism, and Ascorbate and aldarate metabolism were significantly upregulated in transgenic *oHIOMT* line comparing with EV (Supplementary File 2). Furthermore, a total of 166 pathways, with 141 upregulated and 66 downregulated pathways, represented the DEGs between *AANAT* and *HIOMT* groups, including “ribosome,” “oxidative phosphorylation,” “photosynthesis,” “DNA replication” and “calcium signaling pathway.”

### Validation of gene expression profiles using RT-qPCR

**Figure 6:**
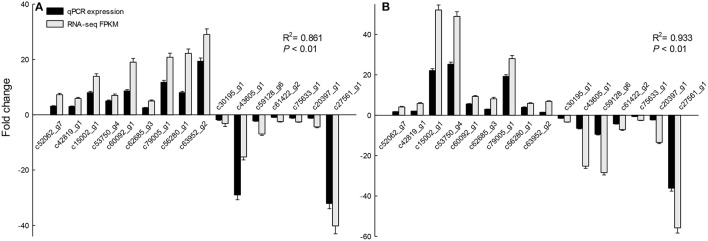
**Validation of RNA-Seq analysis by quantitative real-time PCR (qRT-PCR)**. FPKM (fragments per kilobase of exon per million fragments mapped) values obtained with RNA-Seq and qPCR values in the analysis of selected genes in the three assayed groups: **(A)** transgenic *oAANAT* line, **(B)** transgenic *oHIOMT* line. Error bars represent the standard error for three independent experimental replicates.

The 16 DEGs were selected to evaluate the different expressions for qRT-PCR. Total RNA samples extracted from switchgrass leaves at reproductive stages were used as templates. Histograms were generated by comparing the FPKM determined by transcriptome analysis and qRT-PCR. Expression quantities of the selected genes using RT-qPCR were consistent with the results obtained with RNA-Seq analysis, which means the RNA-seq data were credible. (*R*^2^ = 0.861 for *AANAT, R*^2^ = 0.933 for *HIOMT, P* < 0.01) (Figure 6).

## Discussion

The catalytic reactions of the two key enzymes (AANAT and HIOMT) are the last two steps during the melatonin biosynthesis processes, and the overexpression of the two genes significantly enhanced the melatonin levels in rice (Kang et al., 2010) and tomato (Wang et al., 2014). In our study, the average melatonin contents of transgenic *oAANAT* and *oHIOMT* lines was 12 and 36% (*P* < 0.05) higher than that of EV control, respectively. Melatonin aids plants in terms of root growth, leaf morphology, chlorophyll preservation, and fruit development (Reiter et al., 2015). The enhanced phenotypic growth traits in transgenic *oHIOMT* switchgrass were in accordant with the increasing of the melatonin contents, and average shoot height, leaf length, root number, root length, and spike length of transgenic *HIOMT* lines surpassed those of EV control (Figure 1; Supplementary Table 3). The transcriptional level of the related genes was significantly upregulated, for instance, F-box/kelch-repeat protein, zinc finger BED domain-containing protein 3, RAX2-like protein and so on (Table 1). Other researches demonstrated that increasing of melatonin significantly stimulate vegetative growth by both exogenous and endogenous applications in several plant species (Hernández-Ruiz et al., 2005; Wang et al., 2014).

**Table 1:** **The summary list of differentially expressed unigenes in transgenic ***oHIOMT*** (H), ***oAANAT*** (A), and empty vector (EV) lines**.

**No**.	**Gene_id**	**H vs. EV**	**log2ratio (H vs. EV)**	**A vs. EV**	**log2ratio (A vs. EV)**	**A vs. H**	**log2ratio (A vs. H)**	**Annotation**
1	c43728_g1	U	2.362	F	0.5403	D	−1.814	wound induced proteinase inhibitor WIP1
2	c45282_g1	U	2.381	F	−0.340	D	−2.700	Phage protein C
3	c52718_g1	U	1.698	F	0.459	D	−1.224	glycine-rich domain-containing protein 1
4	c58236_g6	U	2.025	F	0.5410	D	−1.469	copper transporter 1
5	c60917_g13	U	4.696	F	0.706	D	−3.976	late blight resistance protein homolog R1A-6 OS
6	c62742_g1	U	1.351	F	0.301	D	−1.039	SPX domain-containing membrane protein
7	c64524_g1	U	2.476	F	1.112	D	−1.350	glycine-rich domain-containing protein 1
8	c65280_g2	U	1.618	F	0.329	D	−1.278	U-box domain-containing protein 4
9	c56995_g3	U	9.160	D	−3.093	D	−12.245	Acetylserotonin O-methyltransferase
10	c52804_g3	D	−2.202	U	1.583	U	3.794	plasma membrane
11	c46269_g1	D	−2.273	F	−0.125	U	2.159	NAC domain-containing protein 67
12	c58750_g3	D	−2.518	F	−0.360	U	2.169	ATP-dependent RNA helicase DHX36
13	c59344_g7	D	−10.20	F	0.981	U	11.200	VQ motif-containing protein 8
14	c61012_g1	D	−1.754	F	0.443	U	2.206	lectin-like receptor protein kinase family protein
15	c93391_g1	D	−6.149	F	−0.206	U	5.953	structural protein 2
16	c32847_g1	F	NA	U	Inf	U	Inf	protein disulfide-isomerase
17	c33992_g1	F	−0.496	U	3.440	U	3.942	cytochrome c oxidase subunit 3
18	c35379_g1	F	NA	U	Inf	U	Inf	phenylalanine-4-hydroxylase
19	c35977_g1	F	NA	U	Inf	U	Inf	serine protease family S01A
20	c38813_g1	F	NA	U	Inf	U	Inf	SWIB domain-containing protein 1
21	c39636_g1	F	NA	U	Inf	U	Inf	leucine aminopeptidase 1
22	c40464_g1	F	NA	U	Inf	U	Inf	trypsin-like serine protease
23	c41376_g1	F	NA	U	Inf	U	Inf	NADH dehydrogenase subunit 1
24	c44489_g1	F	NA	U	Inf	U	Inf	acid phosphatase
25	c45327_g1	F	NA	U	Inf	U	Inf	heat shock protein 83
26	c47781_g1	F	NA	U	Inf	U	Inf	cytochrome P450
27	c49171_g2	F	0.477	U	1.748	U	1.284	F-box protein At5g51370
28	c49204_g1	F	NA	U	Inf	U	Inf	tyrosine decarboxylase 1
29	c50619_g1	F	NA	U	Inf	U	Inf	ATP synthase F0 subunit
30	c51184_g2	F	0.344	U	1.443	U	1.112	chlorophyll a-b binding protein 8
31	c51629_g1	F	0.020	U	1.278	U	1.265	glucan endo-1,3-beta-glucosidase
32	c52480_g1	F	0.966	U	2.506	U	1.548	galactinol synthase 2
33	c53871_g2	F	0.080	U	1.261	U	1.189	zinc finger protein CONSTANS-LIKE 3
34	c57096_g2	F	0.517	U	1.752	U	1.240	F-box protein PP2-A13
35	c30658_g1	F	−0.304	D	−2.256	D	−1.943	phytosulfokines 5
36	c40152_g1	F	0.738	D	−7.143	D	−7.865	cationic peroxidase SPC4-like isoform X3
37	c46161_g1	F	−0.122	D	−1.485	D	−1.352	Cysteine-rich receptor-like protein kinase 36
38	c50940_g1	F	−0.126	D	−1.370	D	−1.234	ABC transporter G family member 3
39	c53039_g1	F	1.590	D	−2.496	D	−4.072	ent-copalyl diphosphate synthase 1
40	c54359_g2	F	0.978	D	−2.271	D	−3.233	disease resistance protein RPM1-like
41	c54403_g1	F	0.635	D	−2.400	D	−3.026	L-ascorbate oxidase
42	c62341_g2	F	0.140	D	−3.478	D	−3.602	lysine-specific demethylase JMJ25-like
43	c63308_g5	F	−0.818	D	−3.649	D	−2.824	disease resistance protein RGA3

*U, significantly up-regulated (corrected P < 0.05); D, significantly down-regulated (corrected P < 0.05); and F, not differentially expressed (corrected P > 0.05). No. 1–8 and No. 11–15 genes indicated the specific up-regulation and down-regulation in H; No. 9 gene was upregulated in H but downregulated in A line, while No. 10 was oppositely downregulated in H but upregulated in A line; No. 16–34 and No. 35–43 showed the specific up-regulation and down-regulation in A line. NA or Inf presented if the adjusted readcount is zero*.

Furthermore, the transgenic *oHIOMT* switchgrass exhibited earlier flowering than that of the EV, suggesting that the reproductive development was expedited by the endogenous addition of melatonin contents (Figure 1). The reduction and delay of flowering were reported under the exogenous melatonin treatment of *Chenopodium rubrum* (Kolář et al., 2003) and transgenic melatonin-rich rice (Byeon and Back, 2014), respectively. Melatonin is potentially involved in the regulation of flowering process by the similar pattern of the antioxidant ascorbic acid (Kotchoni et al., 2009) or the impediment of the floral transition from vegetative growth to reproductive growth by the repressor of gibberelic acid (GA) pathway (Shi et al., 2016b). However, other researches implied that melatonin was transiently induced with a peak level during flower development in rice (Park et al., 2013). In our study, one of the flowering regulator encoding gene *APETALA2* significantly upregulated in transgenic *oHIOMT* line (log2FoldChange = 6.11 for c52062_g5, *P* < 0.01, Table 1) comparing with the EV control, which acts on the regulation of establishment of the floral meristem (Chen, 2004). The accumulation of melatonin represented the resistance for internal and external oxidative stress during reproductive development (Park et al., 2013).

Melatonin also plays innate immune responses to the complex of biotic and abiotic stresses in various plants species, dicots and monocots (Lee et al., 2014; Shi et al., 2015b; Hardeland, 2016). As summarized in recent publications, increases of melatonin were typically induced by diverse forms of stresses, including extreme temperatures, drought, salinity, and oxidant (Li et al., 2012; Zhang et al., 2014a; Fan et al., 2015). From our results, the overexpression of *oHIOMT* gene also drives a series of defense genes activated, for instance, c43076_g1 gene (annotated to “response to stress”) was significantly upregulated comparing with the EV (Supplementary File 1). In addition, the several amino acid and secondary metabolite related pathways were significantly upregulated, including flavonoid biosynthesis, tyrosine metabolism, beta-Alanine metabolism, glycine, serine, and threonine metabolism, brassinosteroid biosynthesis, phenylalanine metabolism, suggesting the activated growth and stress responses in transgenic *oHIOMT* lines (Supplementary File 2). Other researches exhibited similar tolerance to drought in transgenic *MzASMT A. thaliana* (Zuo et al., 2014), and the resistance to herbicide-induced oxidative damages in transgenic melatonin-rich rice (Park et al., 2012). The transcriptional regulation of defense genes alleviate the oxidative damages driven by various stresses (Wei et al., 2016).

Notably, the differentially expressed unigenes in *HIOMT* vs. EV (405) was less than those of *AANAT* vs. EV (946), suggesting that the overexpression of *oAANAT* could affect more growth-related genes than that of *oHIOMT*. In contrast, the significantly enriched pathways of the DEGs in *HIOMT* were more than those of *AANAT*, indicating that the alterations of involved processes driving by the *O*-methyltransferase were more complex (Figures 3, 5). The previous RNA-seq analysis of overexpression of *oAANAT* gene reported a large number of differentially expressed unigenes, which were majorly involved in various compound biosynthetic processes and organelle component (Yuan et al., 2016). Here, the synchronously upregulated transcriptional factors and growth related genes in transgenic *oAANAT* and *oHIOMT* implied the prompting roles of melatonin in plant growth and development comparing with EV. In addition, a number of differentially expressed genes specifically driving by transgenic o*AANAT* or o*HIOMT* potentially indicated the typical functions of each enzyme. For instance, several stress resistant related genes (SPX domain-containing membrane protein, copper transporter 1, late blight resistance protein homolog R1A-6 OS etc.) were specifically and significantly upregulated in transgenic *oHIOMT* only, while metabolism-related genes (phenylalanine-4-hydroxylase, tyrosine decarboxylase 1, protein disulfide-isomerase, and galactinol synthase 2 etc.) were significantly upregulated in transgenic *oAANAT* only (Table 1). The previous transgenic tomato represented the branching phenotype in overexpressing of *oAANAT* and *oHIOMT* and drought tolerance in transgenic *oHIOMT* lines (Wang et al., 2014). These indicated the both similarity and differences of the functions of the two key enzymes in the melatonin biosynthesis and metabolic networks. Furthermore, several researches regard the AANAT/SNAT is the rate-limiting enzyme during the melatonin biosynthesis (Morton and Forbes, 1988), while the other recent studies consider that the HIOMT/ASMT is the functional one (Byeon et al., 2015, 2016). The previous observation of the nearly two times of leaf melatonin level in *oHIOMT* line of transgenic tomato than those in *oAANAT* lines, provided the proof that ASMT, the homologous to HIOMT, was possibly the rate-limiting enzyme in plants (Wang et al., 2014). Although the similar patterns of the more melatonin accumulation and metabolic pathways were involved in the overexpression of the *oHIOMT* lines than those from the *oAANAT* ones in switchgrass seem to support the HIOMT is the rate-limiting enzyme (Figure 5), the large amount of KEGG pathways related to animal diseases possibly indicated the limited knowledge of melatonin functions in plants through the animal-oriented transgene. Therefore, further investigations from plant-oriented transgenic donor will be expected to provide valuable information for rate-limiting enzyme during the melatonin biosynthetic process.

The multiple membrane receptors and signal transduction mechanism were clarified in animals (Reppert et al., 1994). However, no high-affinity melatonin receptor was identified in plants to date (Park, 2011). Therefore, the receptor-independent mechanism of melatonin in plants was proposed as the neurons (Jan et al., 2011). In our study, the c58996_g3 unigene significantly downregulated (log2FoldChange = −1.768, padj < 0.01), which was annotated as G-protein coupled receptor signaling pathway by GO classification. The discrepancies between increasing melatonin contents and decreasing signaling receptor roughly sustain the hypothesis that melatonin might act via a receptor independent mechanism.

In conclusion, the transcriptomic data from transgenic *oHIOMT* gene switchgrass revealed amount of differentially expressed unigenes comparing with transgenic *oAANAT* and control lines, implying that the last step of catalytic reaction probably is the rate-limiting step during the melatonin biosynthesis in plants. The potential roles of melatonin in plants were indicated by the upregulation of a series of transcriptional factors and functional genes involving growth and resistance for various stresses. Moreover, additional experiments under adverse environmental stresses will confirm the melatonin physiological functions in plants. The definitions of the membrane receptors and signal transduction will largely drive the explanation of the melatonin biosynthesis processes and functional metabolic pathways.

## Author contributions

Conceived and designed the experiments: YZ and FY. Performed the experiments: SY, CG, SL, and YH. Analyzed the data: SY, CG, YH, XC, and DT. Wrote the paper: SY, CG, and YZ. All authors reviewed and approved the final manuscript.

### Conflict of interest statement

The authors declare that the research was conducted in the absence of any commercial or financial relationships that could be construed as a potential conflict of interest.
