# Supplementary material for: Comparative Transcriptomic Analyses of Differentially Expressed Genes in Transgenic Melatonin Biosynthesis Ovine HIOMT Gene in Switchgrass
Source: Front Plant Sci. 2016 Nov 8;7:1613. doi: 10.3389/fpls.2016.01613 (PMC5099686; doi:10.3389/fpls.2016.01613)
Supplement: Supplementary file 3 [file DataSheet3.doc]

**Supplementary Tables**

**Comparative transcriptomic analyses of differentially expressed genes in transgenic melatonin biosynthesis ovine *HIOMT* gene in switchgrass**

**Shan Yuan^1#^, Cong Guan^1#^, Sijia Liu^1^, Yanhua Huang^1,2^, Danyang Tian^1^, Xin Cui^1^, Yunwei Zhang^1,3,4^*, Fuyu Yang^1,5^***

1 College of Animal Science and Technology, China Agricultural University, Beijing, China; 2 College of Agriculture, China Agricultural University, Beijing, China; 3 Beijing Key Laboratory for Grassland Science, China Agricultural University, Beijing, China; 4 National Energy R&D Center for Biomass (NECB); 5 Beijing Sure Academy of Biosciences

**Correspondence and requests for materials should be addressed to Y. Z (Email: zywei@126.com Tel: +86-10-62733572 Fax: +86-10-62733572). F. Y (Email:* [*Yun*](mailto:yfuyu@cau.edu.cn) *Tel: +86-10-010-62733052* *Fax: +86-10-62734252).*

Supplementary Table S1 Oligonucleotide primers used in RT-PCR analysis

| No. | Unigene | Forward primer (5'-3') | Reverse primer (5'-3') |  |
| --- | --- | --- | --- | --- |
| P1 | AP-EREBP | CAACGCTAGGGCAAGGAAA | CATCTCTGATGCCCACGTTT | |
| P2 | SNF | GGTGACTTACCACCAGAACTAAC | GATGACCCGTCGGTTTGATTA | |
| P3 | MYB | CATGCCTGCACCTAACAAATTA | CGTGGTGGAGGTATGTCAAT | |
| P4 | c15002_g1 | GGATAACTCGACCGTGAAGAAC | GAGCAAGAACAGGGAAGAGATT | |
| P5 | c53750_g4 | TGTTCTGGAGCTTCTGGTTATG | GCTTTGGTAAACCGCAAGAAG | |
| P6 | c60092_g1 | CCCGTCAATTCCTTTGAGTTTC | CCTAGCCGTAAACGATGGATAC | |
| P7 | c62685_g3 | CGCTCGATCAGTGAGCTATTAC | CGCTCAGTGATAAAGGAGGTG | |
| P8 | c79005_g1 | GGATCACCTTCTTTCTGGTTCT | TATACGACGGGACTGGTTCT | |
| P9 | c56280_g1 | GTGGAGTAGCTATGGAGGAGTT | CCTATGTCTTGCGCTGTTTCT | |
| P10 | FAR1 | CGACAGAAAGCAGGACATGAA | TTGTTGCTGGATCACGACTG | |
| P11 | c43605_g1 | CACTCCGCCCATGTAAGTAA | GTCGCTACTCGCAAATCTAAAC | |
| P12 | c59128_g6 | CCAGTGTGGAAGACTCAGAAA | GATCTGCCTCCTAATTTGTTCATTC | |
| P13 | c61422_g2 | ATCTAAGCTAGAGGAATCCAACAAA | CATGTTTACTTTAGCCGCGATTT | |
| P14 | c75633_g1 | GAAACCGAGCCCAATCCAT | GTCTTTGCCGGTGGATTCT | |
| P15 | c20397_g1 | GACACGTACGCTCCAGAATAA | GACGGTAGCTCCCATACATTATC | |
| P16 | c27561_g1 | GAAGCCGGAGGACCTTATTC | TTGTGTAGGGTAGGTCCTAGTT | |

Supplementary Table S2 Summary of Illumina transcriptome sequencing. EV: expressing the empty vector only, A: transgenic *oAANAT* line.

| Sample | Raw Reads | Clean Reads | Clean Bases | Error(%) | Q20(%) | Q30(%) | GC Content(%) |
| --- | --- | --- | --- | --- | --- | --- | --- |
| H_1 | 80251080 | 76669864 | 11.5G | 0.01 | 97.47 | 93.64 | 58.51 |
| H_2 | 77435486 | 74289698 | 11.14G | 0.01 | 97.62 | 93.89 | 57.94 |
| H_6 | 84731144 | 80270190 | 12.04G | 0.01 | 97.45 | 93.65 | 58.33 |
| EV_1 | 80891154 | 76976922 | 11.55G | 0.01 | 97.24 | 93.14 | 58.71 |
| EV_2 | 87541130 | 81777632 | 12.27G | 0.01 | 97.22 | 93.26 | 57.87 |
| EV_3 | 89891632 | 84557876 | 12.68G | 0.01 | 97.18 | 93.18 | 55.11 |

Supplementary Table S3 Morphological characterization of transgenic switchgrass plants. EV: expressing the empty vector only, *H*: transgenic *oHIOMT* line.

|  | Tiller number | Plant height  (cm) | Stem node number | Internode length (3) (cm) | Internode  diameter  (cm) | Leaf blade length  (cm) | Leaf blade width  (cm) | Root number | Root length  (cm) | Root diameter (cm) | Spike length  (cm) |
| --- | --- | --- | --- | --- | --- | --- | --- | --- | --- | --- | --- |
| EV | 6.1±0.4a | 66.81±4.15b | 3.2±0.44a | 8.78±0.61b | 2.95±0.23a | 43.53±2.76b | 1.17±0.10a | 16.5±1.35b | 41.95±0.35b | 0.99±0.06b | 5.40±2.31b |
| *H* | 6.7±0.3a | 90.60±6.22a | 3.7±0.28a | 13.43±1.03a | 2.81±0.23a | 52.53±4.45a | 1.09±0.16a | 29.0±1.96a | 49.63±1.91a | 1.38±0.09a | 19.53±2.04a |
